# Role of the Alpha-B-Crystallin Protein in Cardiomyopathic Disease

**DOI:** 10.3390/ijms25052826

**Published:** 2024-02-29

**Authors:** Andres Thorkelsson, Michael T. Chin

**Affiliations:** 1 Tufts University School of Medicine, Tufts University, Boston, MA 02111, USA; andres.thorkelsson@tufts.edu; 2 Molecular Cardiology Research Institute, Tufts Medical Center, Boston, MA 02111, USA

**Keywords:** alpha-B-crystallin, cryab, molecular chaperone, desminopathy, hypertrophic cardiomyopathy, dilated cardiomyopathy, restrictive cardiomyopathy, calcineurin, NFAT

## Abstract

Alpha-B-crystallin, a member of the small heat shock family of proteins, has been implicated in a variety of cardiomyopathies and in normal cardiac homeostasis. It is known to function as a molecular chaperone, particularly for desmin, but also interacts with a wide variety of additional proteins. The molecular chaperone function is also enhanced by signal-dependent phosphorylation at specific residues under stress conditions. Naturally occurring mutations in *CRYAB*, the gene that encodes alpha-B-crystallin, have been suggested to alter ionic intermolecular interactions that affect dimerization and chaperone function. These mutations have been associated with myofibrillar myopathy, restrictive cardiomyopathy, and hypertrophic cardiomyopathy and promote pathological hypertrophy through different mechanisms such as desmin aggregation, increased reductive stress, or activation of calcineurin–NFAT signaling. This review will discuss the known mechanisms by which alpha-B-crystallin functions in cardiac homeostasis and the pathogenesis of cardiomyopathies and provide insight into potential future areas of exploration.

## 1. Introduction

**Figure 1:**
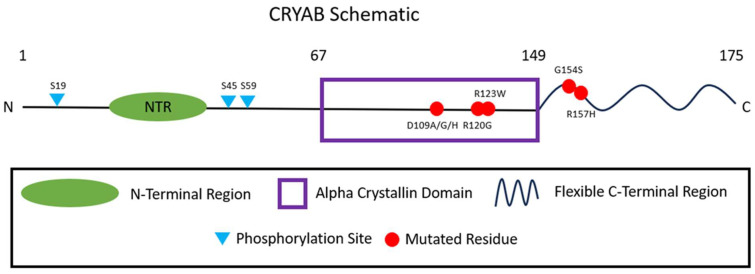
Diagram depicting the domains, mutated residues, and phosphorylation sites of wild-type CRYAB.

Proteins are the molecular effectors of cell function, providing structure and functionality in support of the essential biomolecular processes necessary for organism survival and proliferation. Chaperone proteins are present in a wide variety of organisms across the evolutionary spectrum and function to promote and maintain proper folding of proteins, especially under stress conditions. In response to increased temperatures, and other stressors such as oxidative stress, inflammation, and radiation, organisms initiate a “heat shock response”, probably more aptly named a stress response, based on activation of, and increased expression of, heat shock proteins (Hsps) [1]. Of the many classes of heat shock proteins, the first to be discovered and the most abundantly expressed within cells are the molecular chaperones [1]. Molecular chaperones, divided into groups based on function, help stabilize, fold, and refold proteins even at physiologic temperatures but become even more critical for survival during times of cellular stress [1]. One such group is the small heat shock protein (sHSP) superfamily whose members prevent aberrant protein interactions [1]. Small heat shock proteins function as ATP-independent molecular chaperones that share a common domain architecture across their members [2]. Common domains across the members include a highly variable amino acid N-terminal region, a central alpha-crystallin domain (ACD), and a flexible highly disordered C-terminal region [2]. These structural domains demonstrate distinct behaviors related to their amino acid composition. For example, the abundance of histidine residues in the ACD is thought to allow sHSPs to respond to changes in pH and metal ion availability, which is critical to their proper function [2] (Figure 1). Studies have implicated both the N-terminal region and the ACD as being directly involved in chaperone activities, while the C-terminal region likely plays a supportive structural role necessary for proper chaperone function [2]. As natively folded proteins destabilize, they likely expose hydrophobic residues, which become a signal for sHSP binding and stabilization [2]. The sHSPs, through their ACD, overlap structurally with another distinct set of proteins, the crystallins. The originally described function of crystallins is in the transparency and refractive power of the eye lens, although some have other important cellular functions such as preventing protein aggregation [3]. Crystallins are separated into two groups based on a conserved core domain among the related proteins as either alpha-crystallins or beta/gamma crystallins [3]. The major crystallin groups are categorized into two superfamilies of proteins, the alpha-crystallins which fall under the small heat shock protein superfamily, and interestingly have their own rarely used sHSP designators, and the beta/gamma crystallins which make up their own protein superfamily [3]. The alpha-crystallins are made up of two genes: *CRYAA* and *CRYAB* which encode alpha A and alpha B crystallins, respectively. Due to the ubiquitous nature of molecular chaperones, both these proteins are involved in a myriad of cellular functions and processes [1]. However, this also means that mutations in *CRYAA* and *CRYAB* have a wide array of deleterious effects from cancer to eye disorders and cardiac diseases. Decades ago, a novel *CRYAB* mutation was found to cause hypertrophic cardiomyopathy [4], sparking multiple studies into the effect of *CRYAB* mutations in cardiovascular disease. In this review, we focus on the cellular functions of CRYAB and the broad set of consequences associated with its dysfunction with a particular focus on its role in heart disease, highlighting decades of research and exciting new developments. 

## 2. Alpha-Crystallin B Chain (CRYAB)

### 2.1. Wild-Type CRYAB

Crystallins were initially discovered in the eye lens, where they are the predominant structural protein [5]. From there, more and more crystallins have been found in organs across the body where they serve to prevent improper protein folding and aggregation. Of the two alpha-crystallin members, CRYAA is mainly found in the eye but also in the pituitary gland and spleen, while CRYAB is widely expressed across all organs and is highly expressed in skeletal and cardiac muscle [5]. Most of the beta/gamma crystallins are involved in the transparency and refractive power of the lens; however, some have been found to have other functions, including betaB2-crystallin involved in neurogenesis and betaA3-crystallin involved in calcium binding [5]. Wild-type CRYAB functions as a molecular chaperone, where its main functions are to prevent improper protein folding and aggregation and thus prevent proteotoxicity in cells [6,7,8]. Wild-type CRYAB has also been found to be an anti-apoptotic regulator via multiple pathways such as inhibition of caspase-3 and Ras and inhibition of inflammatory responses, such as decreasing pro-inflammatory peptides and activation of macrophage immunoregulation [9]. Additional functions include the regulation of calcium signaling [10], autophagy [11,12], and cellular survival [13].

CRYAB binds to denatured proteins and enhances their solubility, which plays an important role in preventing protein precipitation in cells [14]. Proteotoxicity is the state in which unfolded and aggregated proteins negatively impact cellular function [15]. Proteotoxicity can be divided into four classes based on functional effects: (1) improper protein folding or structural preservation resulting in altered degradation, (2) poor protein function due to dominant negative mutations, (3) toxic functions due to gain of function mutations, and (4) toxic aggregation of multiple misfolded proteins [15]. Wild-type CRYAB functions to prevent the first and the fourth of the proteotoxic classes from occurring in cells. Improper protein folding is a universal problem that can occur in all cells. Protein folding is in part based on the primary amino acid sequence and is influenced by the amino acid side chains [16]. Side-chain hydrophobicity plays a major role [16]. However, given that the free energy, and therefore the stability, of native proteins is only a few kcal/mol lower than that of their unfolded counterparts, other intramolecular forces, such as backbone hydrogen bonding, cannot be excluded [16]. The relatively small amount of free energy separating folded and unfolded proteins also highlights the fact that even single amino acid mutations can result in consequential changes to protein structure and function [16]. Even though most proteins exist in their minimal free energy, natively folded state, a persistent degree of misfolding and unfolding can occur in cells even without stress. To stabilize proteins and prevent unfolding, one mechanism that cells have developed to mitigate this process relies on the use of molecular chaperone proteins. As a molecular chaperone, wild-type CRYAB plays a major role in preventing aberrant misfolding and guarding against the development of proteotoxicity. Therefore, it is not surprising that wild-type CRYAB is upregulated in a number of cardiovascular disorders, many of which involve some degree of proteotoxicity [13]. It should be noted that a wide variety of proteins and cellular components function to maintain normal protein folding, and mutations that affect these entities lower cellular capacity to maintain proper folding but individually do not fully abolish proper protein folding [15]. Therefore, the emergence of clinically apparent pathology may require long periods of repeated injury and additional stress on the system to provoke pathological changes [15]. 

CRYAB is activated in response to stress through post-translational modification. In response to a number of stresses that can cause alterations in protein folding, both physiological such as heat, TNF-α, and IL-1α and experimental such as okadaic acid and high concentrations of NaCl, CRYAB is phosphorylated at three different serine residues: Ser-19, Ser-45, and Ser-59 [17] (Figure 1). Interestingly, no phosphorylation has been seen in response to agents that increase intracellular cAMP [17]. When phosphorylated, CRYAB translocates from the cytosol to the cytoskeleton presumably to prevent protein destabilization [17]. CRYAB phosphorylation is likely driven by MAP kinase-activated protein 2 which is itself activated by p38 MAP kinase, suggesting its role in the regulation of CRYAB activity, but it could also be driven by p42/p 44 MAP kinase [17]. Studies have shown that wild-type CRYAB overexpression is benign and protective against ischemia and reperfusion injury in vitro and in vivo in transgenic mouse models [18]. Furthermore, cardiovascular diseases are often associated with increased oxidative stress. In that vein, overexpression of wild-type CRYAB in H9C2 cells has been shown to protect against oxidative stress and the apoptosis that accompanies it [19]. The reduction in apoptosis occurs in association with decreased release of cytochrome c from the mitochondria and downregulation of the apoptosis regulator BCL2, which might be mediated through the PI3K/AKT pathway [19]. Wild-type CRYAB is upregulated as an apoptosis inhibitor in certain cancers, and although this article will focus on the cardiovascular system, it is interesting to see the wide range of biological processes influenced by CRYAB [14]. The role of wild-type CRYAB as a molecular chaperone is more fully understood through naturally occurring mutations that result in cardiac pathology, as discussed in the following sections.

### 2.2. CRYAB 109 Mutations

Mutations in the 109th amino acid of CRYAB have been associated with a range of pathologies from cataracts to myopathies [20,21]. In terms of cardiac dysfunction, one of the more common mutations noted is *CRYAB*^D109G^, a missense mutation that has been implicated in the development of restrictive cardiomyopathy [21]. Two additional mutations have been noted at the 109th amino acid: *CRYAB*^D109A^, described by Fichna et al. in 2017 [22], in which patients develop isolated myofibrillar myopathy without cardiac involvement, and *CRYAB*^D109H^, described by Sacconi et al. in 2012 [23], in which a single patient presented with late stage dilated cardiomyopathy [21]. The CRYAB protein spontaneously forms dimers which then form oligomers in physiologic conditions minimizing activity [24,25]; these structures are disrupted in response to stress resulting in its activation and chaperone function [26,27]. The amino acid D109 is highly conserved across species as it forms an integral ionic bridge stabilizing the CRYAB dimer [21], the loss of which appears to lead to aberrant chaperone function. 

The pathology of CRYAB^D109G^ involves abnormal desmin aggregation, based on immunofluorescence localization of these aggregates in C2C12 and Hl-1 cells overexpressing CRYAB^D109G^ [21]. Desmin is a muscle-specific intermediate filament that helps stabilize the contractile apparatus and nucleus in sarcomeres and plays a role in sarcomere architecture. Additionally, desmin plays a role in maintaining tissue structure by tightly associating with cell–cell adhesion complexes [28]. Desmin is highly expressed in muscle tissue, and proper organization of the desmin filaments is key to maintaining cellular function. Cardiac dysfunction often results from disruption of cardiac structure causing an alteration in contractile function; interestingly, CRYAB^D109G^ affects cardiac cell structure indirectly through improper desmin function [21]. 

Pathologies arising from desmin-related dysfunction and aggregation are termed desminopathies, and when they involve muscle tissue, they are named desmin-related myopathies. Desminopathies can arise from mutations within desmin itself, and several pathogenic desmin mutations have been described; however, they can also arise from the dysfunction of proteins involved in protein folding and stability [28]. CRYAB^D109G^ results in the development of desmin-related cardiomyopathy because the mutant CRYAB is no longer able to efficiently stabilize and prevent the aggregation of desmin filaments. Desmin aggregation in cells is broadly characterized by two criteria defined by Goebel [29], (1) multifocal cytoplasmic inclusions or spheroid bodies and (2) disseminated accumulation of granulofilamentous material [28]. Wild-type CRYAB forms stable dimers through ionic bridges between D109 and R120, which are disrupted by mutations in the region and are a particularly common site of missense mutations in patients with myopathies [21]. Therefore, instead of binding desmin to stabilize the Z-bands and intercalated disks in muscles, they form cytoplasmic aggregates in conjunction with the mutated CRYAB protein, falling into the first classification of desmin aggregation [21]. When the desmin filaments then aggregate, they cause cellular dysfunction which in the heart manifests mostly as forms of cardiomyopathies both hypertrophic and restrictive, although hypertrophy is more commonly noted [21]. 

### 2.3. CRYAB 120 Mutations

Mutations at the 120th amino acid of CRYAB, like mutations at the 109th amino acid, are also involved in various pathologies across the body. The most common mutation associated with cardiovascular disease is the germline *CRYAB*^R120G^ missense mutation, which is inherited in an autosomal dominant manner [30]. As was noted in the previous section, CRYAB forms dimers that are stabilized by ionic bonds at the D109 and R120 amino acids [21]. Interestingly, cryoelectron microscopy of purified CRYAB^R120G^ has shown an abnormal quaternary structure with a molecular weight at least twice that of wild-type CRYAB, suggesting the mutation facilitates abnormal oligomerization [31]. Interestingly, in vitro studies indicate that CRYAB^R120G^ acts in a dominant negative manner, with the mutant protein compromising the function of wild-type proteins in the dimerized form [18]. CRYAB mutant aggregation then suggests that even in the cases of heterozygous mutations in CRYAB, the mutant protein might cause wild-type proteins to form aggregates resulting in the development of cardiac pathology. As was seen in the mutations at D109, mutations at R120 also lead to desmin aggregation and subsequent cellular dysfunction with loss of normal muscular striations seen in cardiomyocytes isolated from CRYAB^R120G^ transgenic mice [30]. Desmin-related myopathies can be defined based on electron-dense granular aggregates in the cytoplasm seen in electron microscopy [30]. These structures are divided into two classes by Wang et al.; Type I structures had a relevantly low electron density, were large and regularly shaped, and tended to occupy a large portion of the central part of the cardiomyocyte while Type II structures were composed of finer and smaller granules that are more numerous than Type I granules, irregularly shaped, and surrounded by many fine filaments [30]. As was noted in the previous section on CRYAB^D109G^, desmin aggregations can broadly be classified based on appearance as was done by Goebel [29], while the types outlined by Wang et al. are specific for the electron microscopy appearance in desmin-related myopathies. While distinct, the two classification systems correspond to each other as follows: Wang Type I aggregates in electron microscopy correspond to Goebel multifocal cytoplasmic inclusions or spheroid bodies, while Wang Type II aggregates correspond to the Goebel disseminated granulofilamentous material. It appears that Type I granules were mainly composed of mutant CRYAB aggregates, while Type II aggregates were composed of CRYAB mutants and desmin filaments [30]. Although some aggregates contained both desmin and CRYAB mutant protein, interestingly, it was most common for CRYAB and desmin to aggregate independently of the other protein [30]. 

Mice overexpressing the CRYAB^R120G^ variant additionally are under reductive stress, with myopathic hearts showing increased recycling of oxidized glutathione to reduced glutathione due to augmented expression and enzymatic activity of glucose-6-phosphate dehydrogenase (G6PD), glutathione reductase, and glutathione peroxidase [32]. Crossing of these mice with mice expressing reduced levels of G6PD rescued the cardiomyopathic and proteotoxic phenotype [32]. In cells with the CRYAB^R120G^ mutation, autophagy, a process by which dysfunctional cellular components are removed, is inhibited, suggesting another mechanism by which mutant CRYAB negatively impacts the function of cells [33]. Autophagy as a whole can be broken down into three broad categories: (1) macroautophagy, where cytoplasmic contents are sequestered in an autophagosome that then combines with a lysosome for degradation; (2) microautophagy, in which the lysosomal membrane invaginates, engulfing targets of degradation; and (3) chaperone-mediated autophagy, where chaperones target proteins with a specific peptide sequence which is then unfolded and translocated to the lysosome for degradation [34]. In general, autophagy occurs at a base level in the cell, recycling cellular material, particularly damaged proteins, to prevent their harmful accumulation. During stress, increases in autophagy protect the cell from additional harmful accumulation of cellular material, to maintain proteostasis [34]. Furthermore, inducing autophagy in CRYAB^R120G^ cultured cardiomyocytes reduces the aggregation burden and cytotoxic aggregation intermediates, referred to as pre-amyloid oligomers [33]. A previous study observed that in the hearts of mice overexpressing CRYAB^R120G^, there is increased autophagy as an adaptive response to proteotoxic aggregates [11]. Crossing these mice with mice deficient in autophagy due to Beclin deficiency resulted in worsened proteotoxicity and cardiomyopathy [11]. Enhancement of autophagy is thus a viable strategy for improving CRYAB^R120G^-induced proteotoxicity and cardiomyopathy [11]. It is important to note that although protein aggregates are the hallmark of desmin-related cardiomyopathy, their accumulation is only weakly correlated with disease severity, while the amount of pre-amyloid oligomers more strongly correlates with human cardiovascular disease [33]. As was noted in the D109 mutants, the cardiac pathology that is most often associated with CRYAB^R120G^ is the development of desmin-related cardiomyopathy.

### 2.4. CRYAB 123 Mutation

A recently identified mutation in CRYAB by Maron et al., the *CRYAB*^R123W^ mutation, was discovered through genetic analysis in twins that developed hypertrophic cardiomyopathy with temporal concordance [35,36]. Follow-up mouse studies revealed that, unlike the previous two mutations, CRYAB^R123W^ does not cause desmin aggregation but rather leads to cardiac dysfunction through sarcomere-independent mechanisms [36]. Knock-in mice with the CRYAB^R123W^ mutation do not develop hypertrophic cardiomyopathy spontaneously but undergo a distinct remodeling process upon pressure overload via transverse aortic constriction [36]. Wild-type CRYAB has been previously reported to play a protective role against the development of pathological hypertrophy in pressure-overloaded hearts [10]. As for the mechanism behind the protective effects of wild-type CRYAB in this setting, it has been proposed that CRYAB prevents the interaction between calcineurin and NFAT and inhibits the subsequent downstream activation [36]. The CRYAB^R123W^ mutant is unlikely to block that interaction as efficiently, therefore leading to aberrant activation [36]. Crossing of *Cryab*^R123W^ mice with NFAT-luciferase reporter mice resulted in an increase in NFAT-luciferase reporter activity, while overexpression in H9c2 cells also led to increased NFAT-luciferase reporter activity [36]. 

**Figure 2:**
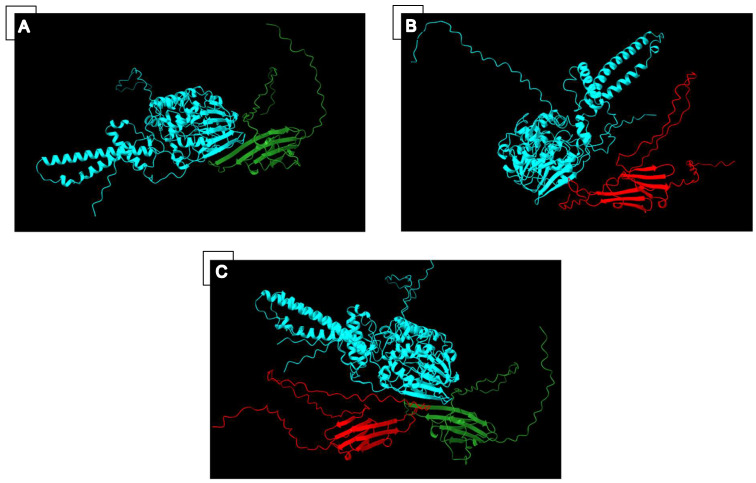
(**A**) Alphafold structural prediction of the interaction between calcineurin (blue) and wild-type CRYAB (green) illustrating that wild-type CRYAB binds well at the calcineurin NFAT binding site. (**B**) Alphafold structural prediction of the interaction between calcineurin (blue) and CRYAB^R123W^ (red) illustrating that CRYAB^R123W^ binds poorly at the calcineurin NFAT binding site. (**C**) Overlap of the previous two Alphafold predictions showing the difference between wild-type CRYAB (green) and CRYAB^R123W^ (red) binding to the calcineurin (blue) NFAT binding site.

Five NFAT transcription factors have been discovered; NFATc1-c4 are regulated by calcineurin, whereas NFAT5 resides in the nucleus and is not under calcineurin regulation. Calcineurin is a serine/threonine phosphatase activated by sustained high levels of calcium that bind to calmodulin and lead to a conformational change in which the calcineurin C-terminal autoinhibitory domain is disengaged. Once active, calcineurin binds NFAT and de-phosphorylates several serine motifs in the regulatory domain of NFAT, exposing its nuclear localization signal leading to its nuclear localization and transcription factor activity [37]. For proper signaling, the calcineurin catalytic domain must be able to bind to the conserved PxIxIT motif on NFAT, located N-terminal to its phosphorylation sites; inability to do so results in NFAT repression [37]. It is possible that wild-type CRYAB blocks this interaction, as it has been shown that wild-type CRYAB inhibits the activation of NFAT and its nuclear translocation [38]. Furthermore, structural analysis by Alphafold multimer, as seen in Figure 2, predicts that wild-type CRYAB strongly occupies the NFAT binding domain of calcineurin while the CRYAB^R123W^ mutant does not. CRYAB^R123W^ would thus be expected to bind less efficiently and facilitate calcineurin/NFAT activation through a de-repression mechanism. Interestingly, however, overexpression of CRYAB^R123W^ in H9c2 leads to activation of NFAT activity, despite the presence of WT CRYAB, suggesting that an activation mechanism is present rather than a simple de-repression mechanism [36].

### 2.5. CRYAB G154S Mutation

*CRYAB*^G154S^ was discovered by Pilotto et al. in 2006 in a 48-year-old female patient found to have dilated cardiomyopathy, without ocular manifestations, with a family history of dilated cardiomyopathy found in her father [39]. The phenotype was characterized by mild LV dilatation, moderately decreased ejection fraction, and a mild increase in serum CPK suggesting possible subclinical muscle involvement [39]. It has furthermore been described by Reilich et al. in 2010 as a cause of progressive late-onset distal myopathy, without cardiac and ocular involvement [40]. Muscle cells were found in histology to be consistent with myofibrillar myopathy with aggregates staining positive for desmin and CRYAB, although the morphology of the aggregates in electron microscopy is different than those reported for *CRYAB*^R120G^ mutations [40]. 

### 2.6. CRYAB R157H Mutation

The *CRYAB*^R157H^ mutation was first noted in 2006 by Inagaki et al.; a 71-year-old patient was found to have dilated cardiomyopathy as well as a family history of dilated cardiomyopathy and sudden cardiac death [41]. Furthermore, CRYAB^R157H^ was found to have an impaired ability to bind to the heart-specific N2B domain of titin/connectin compared to wild-type CRYAB [41]. Since wild-type CRYAB has been found to associate with the I-band region of titin/connectin, it has been suggested that impaired localization of mutant CRYAB^R157H^ to the I-band region predisposes to early progression of heart failure under stressful conditions [41]. Unlike CRYAB^R120G^, CRYAB^R157H^ does not seem to form cytoplasmic aggregates and does not lose affinity for the I26/I27 domain of titin/connectin found in muscle, suggesting a mechanism for the presence of cardiac but not skeletal pathology [41]. Structural analysis of CRYAB^R157H^ found that there was minimal change in the secondary and tertiary structure of the protein; however, there was a significant change in the quaternary structure, with CRYAB^R157H^ forming smaller oligomers upon heat stress [42]. Interestingly, although the mutant protein had lower thermal stability, it maintained a comparable chaperone activity compared to wild-type CRYAB [42]. Considering both the changes in quaternary structure and the maintenance of chaperone activity, it is possible that CRYAB^R157H^ has significant changes in interaction patterns which might play a role in its pathogenesis [42].

## 3. CRYAB Mouse Models

Cardiomyopathies have been studied in various cellular and animal models. Mice are the most common model and are often used in conjunction with genetic alterations and induction of cardiomyopathic phenotypes via additional, often surgical, interventions [43]. Mice have been extensively used because of the ease of genetic modification and animal maintenance; however, they do not always recapitulate the key features of human disease [43]. The use of large animal models of cardiomyopathies, including cats, dogs, and pigs, is growing due to their ability to recapitulate critical features of human physiology and disease, but they have longer life cycles and are more difficult to maintain [43]. Here we will discuss the mouse models that have been generated and used to study CRYAB-related cardiovascular diseases, as current CRYAB research relies almost exclusively on them.

### 3.1. CRYAB R120G Mouse Models

Wang et al. reported the construction of transgenic mice expressing three different expression levels of the CRYAB^R120G^ mutant [30]. Germline transmission was confirmed with normal Mendelian offspring ratios indicating no embryonic lethality across the expression levels [30]. Protein analysis of the transgenic mutant hearts showed elevated levels of proteins, especially of insoluble proteins likely representing protein aggregates seen on stained myocardial sections [30]. As the mutant mice aged, the number and size of aggregates increased [30]. Higher expression of mutant CRYAB^R120G^ increased mortality, indicating a possible dose-dependent phenotype [30]. Mice with the highest expression level died around age 5–7 months, while mice with intermediate expression levels showed a similar phenotype at age 12–16 months [30]. Extracted hearts were grossly enlarged and dilated [30]. Necropsy also revealed pulmonary and hepatic congestion, pleural effusion, and subcutaneous edema consistent with congestive heart failure [30]. The mutant line 708, whose expression was intermediate, and mutant line 134, whose expression was the highest, were chosen for further study and compared to mice expressing transgenic wild-type CRYAB with expression and protein levels comparable to the mutants [30]. On a molecular basis, activation of the fetal genetic program was observed, with an upregulation of atrial natriuretic peptide and β-myosin and a downregulation of α-myosin, phospholamban, and sarcoplasm reticulum calcium in young mice harboring the CRYAB^R120G^ mutant [30]. By 3 months, hypertrophy was grossly apparent based on increased ventricular weight/tibial length ratios and continued to worsen as the mutant mice aged [30]. Cardiomyocyte size progressively enlarged, and at 3 months, the increased heart size was attributed to concentric hypertrophy [30]. However, as the mice aged, the increased size was due to heart dilation suggestive of failure [30]. Both the early molecular changes and physiologic progression were consistent with the clinical progression seen in human cardiovascular diseases [30]. Comparable to the human pathophysiology of desmin-related cardiomyopathy, 3-month-old CRYAB^R120G^ mice maintained contractile function, but relaxation impairments were noted [30]. With age, however, there was progression to severe disease comparable to that in humans with loss of contractile function and relaxation becoming load-dependent [30]. It was noted that total cellular levels of CRYAB^R120G^ increased as the transgenic mice aged, while total levels of wild-type CRYAB in transgenic mice remained constant despite higher transcript levels [30]. This suggests that high levels of wild-type CRYAB are not necessarily detrimental to the cell; however, progressive accumulation of mutant CRYAB^R120G^ protein that aggregates and induces desmin aggregation leads to progressive cardiac damage [30]. Furthermore, it was also found that in CRYAB^R120G^ aggregates, there was often a lack of desmin, suggesting an inability of the mutant to properly bind to desmin. Therefore, desmin aggregates are likely not due to an aberrant interaction with CRYAB^R120G^ but rather form due to loss of chaperone activity [30]. It was also found that desmin null CRYAB^R120G^ transgenic mice have a less severe phenotype compared to mice with intact desmin, which suggests that the pathophysiology is not solely driven by loss of desmin function [30]. A knock-in mouse model expressing normal levels of CRYAB^R120G^ also demonstrates lens and myopathy phenotypes [44].

### 3.2. CRYAB R123W Mouse Models

Mice harboring the *Cryab*^R123W^ mutation were generated by Chou et al. using C57BL/6 mice with CRISPR/Cas9-mediated homology-directed repair to knock in the mutant allele [36]. In this model, mice did not develop hypertrophic cardiomyopathy at a steady state, which is not unexpected given that many models require additional stress for pathology to emerge [36]. At a steady state, young mice homozygous for the *Cryab*^R123W^ mutation were found to have increased E_max_, a load-independent measure of contractility, compared to wild-type and heterozygous mice; interestingly, this seems to decrease with age [36]. Steady-state mice were also found to have an elevated E/E’ indicative of diastolic dysfunction commonly seen in hypertrophic cardiomyopathy patients that developed with age [36]. However, using this model in combination with transverse aortic constriction resulted in the development of marked pathological hypertrophy in homozygous and heterozygous *Cryab*^R123W^ mutants not seen in wild-type mice [36]. Like other mouse models of hypertrophic cardiomyopathy (HCM), these mice developed circumferential hypertrophy as opposed to the asymmetric septal hypertrophy seen in humans [36]. But otherwise, *Cryab*^R123W^ mutant mouse hearts showed a greater extent of cellular hypertrophy and large areas of parenchymal fibrosis compared to the wild type, which was consistent with key features of human HCM [36]. It should also be noted that mice carrying the *Cryab*^R123W^ mutation developed progressive systolic dysfunction after transverse aortic constriction, which did not worsen in mice with both *Cryab*^R123W^ mutation and heterozygous MYBPC3 truncation, suggesting that CRYAB^R123W^ acts in a sarcomere-independent manner [36]. Overall, the *Cryab*^R123W^ mutant mice displayed key elements of human HCM pathology and were stable during steady-state conditions, indicating that these mice are easy to maintain and readily induced to develop pathological hypertrophy with the addition of pressure overload [36]. Of note, however, these mice did not develop proteotoxic desmin or CRYAB aggregates and demonstrated increased calcineurin/NFAT activation, indicating a distinct mechanism of promoting pathological hypertrophy compared to the CRYAB^R120G^ variant [36].

## 4. Therapeutic Approaches

CRYAB-associated cardiac pathology results from the failure of normal protein functions. In the case of CRYAB^D109G^ and CRYAB^R120G^, the driving pathological mechanism is the induction and accumulation of misfolded proteins in the cell resulting in proteotoxicity [21,30]. The CRYAB^R120G^ mutation has been found to result in the development of desmin and aggresome protein aggregates in the cell, likely due to the loss of the molecular chaperone functions that prevent misfolded protein aggregation in response to stress [45]. Interestingly, it has been found that the desmin aggregates associated with CRYAB^R120G^ are amyloidophilic, and the most accurate description of these aggregates then would be amyloid-like. This means that CRYAB-based aggregates share some similarities with other amyloid-based degenerative diseases, such as Alzheimer’s disease, although there is emerging evidence that this is not unique to CRYAB-based cardiomyopathy, as these aggregates have also been found in the hearts of patients with non-CRYAB mutation-induced hypertrophic and dilated cardiomyopathy [45]. As a brief overview of cardiomyopathic disease, from a pathophysiologic standpoint, there are three broad classes: (1) dilated cardiomyopathy is the most common, defined by left ventricular dilation and reduction in ejection fraction; (2) hypertrophic cardiomyopathy, defined by impaired left ventricular relaxation and filling due to thickened ventricular walls; and (3) restrictive cardiomyopathy, defined by decreased elasticity of the myocardium which leads to impaired ventricular filling without systolic dysfunction. CRYAB-based development of cardiomyopathies is summarized in Table 1, and it varies between and even within specific mutations, but CRYAB^D109G^ and CRYAB^R120G^ are associated with desmin-related cardiomyopathies [21,30], while the development of hypertrophic cardiomyopathy due to CRYAB^R123W^ could be related to abnormal calcineurin–NFAT signaling [36]. 

Since the formation of aggregates plays a central role in the increased proteotoxic state associated with desmin-related cardiomyopathy, it stands to reason that preventing or reversing the protein aggregation either by resolubilizing the aggregates or increasing their degradation could help alleviate the disease. Misfolded proteins are targeted and then cleared by the ubiquitin protease system, a central mechanism for minimizing proteotoxicity [46]. Inadequate proteasome function and the resultant increase in proteotoxic stress have been implicated in various human heart diseases and their progression to heart failure [46]. Studies have shown that cGMP-dependent protein kinase stimulates proteasome activity, thereby improving the degradation of misfolded proteins in cardiomyocytes [46]. Cyclic nucleotide phosphodiesterase (PDE) is a key mediator in the breakdown of cGMP and affects the regulation of its associated signaling pathways. Studies have shown that inhibition of PDE1 has protective effects against isoproterenol-induced myocardial hypertrophy and fibrosis in mice and that the depletion of PDE1C is protective against pressure-overload-induced remodeling of the heart via PKA [46]. Transgenic mice expressing mutant CRYAB^R120G^ had a significant elevation in the levels of PDE1A [46]. Furthermore, inhibition of PDE1A in mice expressing CRYAB^R120G^ that had developed heart failure with preserved ejection fraction improved cardiac diastolic function and survival compared to non-treated mice [46]. It should also be noted that PDE1A inhibition resulted in decreased levels of misfolded CRYAB^R120G^ [46]. From a mechanistic standpoint, it has been suggested that PDE1 inhibition improves cardiac function in proteotoxic states through PKA- and PKG-mediated proteasomal activation [46], suggesting that inhibition of PDE1 is a possible therapeutic that can be used to target protein-aggregation-based cardiomyopathies.

Another therapeutic option would be to target protein stability via increased chaperone activity with the intent to prevent or reverse protein misfolding and aggregation, rather than increasing protein degradation. A novel molecular tweezer, CLR01, functions as a nanochaperone, preventing abnormal protein aggregation by selectively binding to lysine residues [47]. The binding of CLR01 to lysine residues is achieved by hydrophobic and electrostatic interactions that compete for binding at critical residues involved in the aggregation of misfolded proteins [47]. Importantly, as a possible therapeutic approach, CLR01 has been tested in several in vitro and in vivo models without signs of toxicity [47]. In transgenic mice expressing CRYAB^R120G^, daily injection of CLR01 resulted in decreased levels of aggregates and improved proteotoxicity in hearts compared to untreated mice [47]. Preventing protein aggregation then could allow misfolded proteins to remain soluble and then remain a target for degradation pathways [47]. Outlined above are two mechanisms that can be targets for therapeutics, increasing misfolded protein degradation and preventing misfolded protein aggregation. Although these are distinct pathways, interestingly, there seems a possibility for synergy between the two methods, with CLR01 increasing the number of misfolded proteins accessible to the protein degradation system and PDE1A inhibition enhancing the efficacy of the proteasomal activation. 

Another example of a possible therapeutic that has been studied is doxycycline, which has been found to improve mortality in CRYAB^R120G^ transgenic mice with late-stage cardiomyopathy [48]. It was found that doxycycline can prevent aberrant protein aggregation in mice with CRYAB^R120G^ desmin-related cardiomyopathy, but interestingly, it does so through an autophagy-independent mechanism as opposed to the previously discussed therapeutics [48]. It was found that there was a decrease in both aggregates and oligomeric CRYAB^R120G^ with doxycycline treatment, suggesting doxycycline inhibits CRYAB^R120G^ from inducing aberrant oligomerization, possibly allowing for normal CRYAB oligomerization [48]. Additionally, it has been found that sHSPs often exist in complexes with other sHSPs or target proteins [49]. HSPB1 and HSPB8 are two other sHSPs able to modify the aggresomal formation of CRYAB^R120G^ and inhibit its ability to induce the formation of amyloid oligomers [49]. This suggests that induction of other non-mutated sHSPs could have therapeutic purposes in CRYAB^R120G^ cardiomyopathy, as was seen when transgenic CRYAB^R120G^ mice were treated with geranylgeranylacetone, an inducer of sHSPs, showed improved survival and heart function and improvements in heart size and fibrosis [49]. Exercise has been found beneficial in delaying the onset and progression of neurodegenerative diseases in animal models, including amyloid-based Alzheimer’s models [49]. Given that some CRYAB mutations induce amyloid-like aggregations, it was hypothesized and discovered that exercise improves both symptoms and mortality in mice with CRYAB^R120G^ cardiomyopathy with a reduction in amyloid oligomers [49]. Cellular death secondary to toxic aggregate formations found in mutant CRYAB cardiomyopathies is another avenue being explored for therapeutics [49]. Studies have found that overexpression of BCL2 and administration of the mitoK(ATP) channel opener to CRYAB^R120G^ mice lead to improvements in mitochondrial function, cardiac function, and survival [49]. These approaches to proteinopathy-based cardiac disease are exciting as they offer various novel methods of therapeutics for a disease that sorely lacks effective medications.

## 5. Conclusions and Future Directions

A large body of evidence has accumulated in support of the essential role of α-B-crystallin in normal cardiac homeostasis through its function as a molecular chaperone to reduce proteotoxic aggregation and to attenuate pathological calcineurin/NFAT signaling. Naturally occurring mutations that lead to desmin-related cardiomyopathy, restrictive cardiomyopathy, and hypertrophic cardiomyopathy underscore its relevance to human disease. An analysis of the pathological mechanisms in these various conditions underscores the broad effects of CRYAB on cellular function and how different mutations can have distinct effects on either protein aggregation or calcineurin/NFAT signaling to promote divergent phenotypes. Future work to determine the specific effects of pathological mutations on CRYAB structure, function, and interacting proteins will likely provide further insight into downstream pathological mechanisms and identify future targets for therapeutic intervention.

## Figures and Tables

**Table 1:** Pathology associated with different CRYAB mutations.

CRYAB Mutation	Associated Pathology	Source
CRYAB^D109G^	Hypertrophic and Restrictive Cardiomyopathy	Brodehl et al., 2017 [21]
CRYAB^D109A^	Isolated Myofibrillar Myopathy	Fichna et al., 2017 [22]
CRYAB^D109H^	Dilated Cardiomyopathy	Sacconi et al., 2012 [23]
CRYAB^R120G^	Desmin-Related Cardiomyopathy	Wang et al., 2001 [30]
CRYAB^R123W^	Hypertrophic Cardiomyopathy	Maron et al., 2020 [35]
CRYAB^G154S^	Isolated Dilated Cardiomyopathy and Late-Onset Distal Myopathy	Pilotto et al., 2006 [39], Reilich et al., 2010 [40]
CRYAB^R157H^	Dilated Cardiomyopathy	Inagaki et al., 2006 [41]

## Data Availability

Not applicable.
